# Navigating care: understanding cancer patients’ experiences with systemic radionuclide therapy

**DOI:** 10.1007/s00520-025-09380-2

**Published:** 2025-03-27

**Authors:** Johannes Starkbaum, Solenn Thircuir, Florian Winkler, Erich Griessler

**Affiliations:** 1 Institute for Advanced Studies (IHS), Josefstädter Straße 39, 1080 Vienna, Austria; 2 ESA Business School, Beirut, Lebanon

**Keywords:** Radionuclide therapy, Neuroendocrine tumor, Patient engagement, Care

## Abstract

Neuroendocrine tumors (NETs) are widely considered to be a rare disease, often diagnosed at a late stage due to the variety of symptoms. Systemic radionuclide therapy (SRT) with Lutathera (177Lu-Dotatate) is a promising treatment for this disease. However, little is known about patients’ experiences with this approach and the radioactivity involved. Based on seven semi-structured interviews, this qualitative study explores how cancer patients perceive their journey to SRT, and the treatment and care they receive in clinics specialized in the delivery of 177Lu-Dotatate to target tumors. The interviews were conducted in France between 2020 and 2021. Six interviews included patients receiving SRT, and one was held with a patient representative for NETs. Three key themes emerged from the analysis: challenges in accessing SRT, including geographic and institutional barriers; the psychological and social impact of radiation-related isolation; and the role of patient-centered care in fostering trust and well-being. Patients reported difficulties navigating the healthcare system, emotional distress due to isolation during treatment, and the importance of support in mitigating these challenges. These findings highlight the need for increased access to SRT, as well as of patient-centered approaches to the various challenges directly and indirectly associated with SRT.

## Introduction

Neuroendocrine tumors (NETs) are rare malignancies arising from neuroendocrine cells [1]. While they may develop in nearly every organ, NETs of gastroenteropancreatic and lung origin represent the most common manifestations. They stand out because of slow disease development, incurability (in most cases), and side-effects of treatment that are different from those of most other cancer types [2]. Surgery is the only curative therapy but often not possible due to late diagnosis. Advanced disease is subject to a variety of individualized systemic treatments options, including targeted therapies such as somatostatin analogues or radionuclide therapy.

Systemic radionuclide therapy (SRT), in which the radioactive substance Lutathera (177Lu-Dotatate) is injected into the patient’s body to destroy tumor cells, has emerged as a promising therapeutic approach, even referred to as a “magic bullet” [3]. However, critical research on innovation in cancer medicine highlights the many stories of disappointment, the uneven distribution of benefits, and the complexities of the involved expectations of care [4]. While knowledge on technical and application-oriented aspects of SRT is increasing, little is known about perspectives of patients who receive this treatment.

Patient empowerment and considering their perspectives in innovation, including medical innovation, is a long-standing call from researchers from social sciences and Science and Technology Studies (STS) in particular [5]. Related research has explored how patient groups link medicine and practice-experiences to better align research with their needs [6]. In line, some scientists have called for patient-centered care and shared decision-making, thus involving patients in advocating for their health [7]. Other studies show how patients and activists have actively engaged with experts to bring their knowledge to relevant medical institutions [8].

Care is a concept used in STS for reflecting on the relationships of patients with others [9]. It extends beyond nursing to include all practices related to treatment, the technologies involved, and the needs and experiences of patients and caregivers. Several empirical studies explore practices in health related to care, e.g., in the context of quality of life of patients [10], the (medication related) care within hospitals [11], or information-seeking with chronic diseases [12]. Care in STS goes beyond the medical context and is inspired by critical and feminist contributions that have highlighted the multifaceted, contextual, and political character of it [13]. As such, it has been described as “an affectively charged and selective mode of attention that directs action, affection, or concern at something, and in effect, it draws attention away from other things” [14, p. 635]. In addition, care in the context of research-intensive emerging technologies such as SRT is subject to the ambiguity of simultaneously accounting for the patients being treated and the patients envisioned by research and innovation activities [4]. Indeed, our research shows that this approach poses several challenges for patients, which calls for the consideration of their perspectives.

Our research originates from the collaborative research project POPEYE,^1^ in which a multidisciplinary team of researchers from the natural and social sciences explored not only the technological advancement of SRT but also studied related Ethical Legal and Social Aspects (ELSA) of it. During the latter, we conducted seven semi-structured interviews in French clinics with NET-patients and one patient representative. Additionally, we did on-site observations at a French clinic that offers SRT, which also included conversations with additional NET-patients. The 177Lu-Dotatate treatment has progressively been implemented within French nuclear medicine departments since it was approved in 2017. However, as it requires authorizations, a specific protocol, management, and settings, this treatment remains a niche and access for patients is limited [15].

This article thus investigates the experiences of patients with NETs in France who were treated with SRT. We explore their perceptions of their journeys towards specialized clinics and their experiences with care at these.

## Research on patients with neuroendocrine tumors

Although their worldwide incidence is rising [16, 17], neuroendocrine tumors (NETs) are still mostly considered a rare disease [18–20]. The classification as rare disease has implications for NET patients and is therefore a central theme discussed on the websites of several NET foundations [21, 22]. Even though Gosain et al. [23] have shown that NET patients in the USA in recent years received an earlier diagnosis, late diagnosis remains a problem for this group of patients, a problem shared with many rare diseases [24].

There is also much learn from more general literature on rare diseases and the challenges arising from their scarcity, including the need to improve timely diagnostic, (equal) access to appropriate healthcare services at local and global scale, and limited (clinical) guidelines and peer support [6, 25]. Other studies highlight the scarcity of resources, scattered expertise, and a lack of appropriate care delivered to patients for rare diseases [26, 27].

However, NETs create significant challenges for affected patients, their families, and clinicians attempting to achieve a confirmed diagnosis and implement the best care. Symptoms of NETs are often non-specific [24, 28], more chronic than the ones of other cancer types and go on for years after diagnosis [16]. Patients are confronted with symptoms such as diarrhea, abdominal symptoms, and flushing [19]. Research emphasizes that NETs have serious impacts on a patient’s quality of life, referring to disease related stress, social isolation, and stigmatization [29–31].

NETs are often understood in similar ways as other forms of cancer, even though they differ from them in significant ways. Plage et al. [2, p. 154] show how “contemporary representations of cancer” affect NET patients and how they deal with a tumor that behaves differently than what is commonly imagined to be cancer. Tropes like the “battle narrative” [2, p. 160], in which a person can become cancer-free if they keep on *fighting* against cancer, seem out of place [28]. NETs are often incurable, and patients must live with them [19]. In a large study on NET patients in the USA, Dasari et al. [32] calculated an overall median survival of 9.3 years after diagnosis, but overall survival depends upon the site and stage of the tumor. For example, people with localized NETs have a median overall survival of more than 30 years [32].

Because NET patients face problems that are different from those of patients with more common cancer types, patient organizations and researchers alike are calling for special measures that are tailored to these needs [22, 28]. Feinberg et al. [28] have shown how patient support programs are designed for more common forms of cancer. Moreover, Feinberg et al. [28, p. 543] highlighted how NET patients have expressed “the need for support specific to their cancer” and that the specific needs of NET patients make it hard for them to find appropriate care. Often, NET patients do not have access to key diagnostics and therapeutics [24]. A recurring argument in the extant literature is that specialist centers have a positive impact on the outcomes and care for these patients [23, 24, 33]. Furthermore, these centers can then facilitate an environment in which health care providers are “more knowledgeable about the rare disease” [28, p. 544].

Surprisingly, contributions on patient experiences with NETs rarely address how SRT and its treatment cycles affect patients’ life and wellbeing. This treatment is merely mentioned as something that patients would like to have more access to [24]. Marinova et al. [34] found that this treatment has a positive effect on health-related quality of life of patients.

## Methods

Between January 2021 and June 2022, we conducted seven interviews to explore perspectives of patients. The interviews were conducted with patients in hospitals specialized on the delivery of 177Lu-Dotatate in three French cities. Of the seven interviews, six involved patients receiving 177Lu-Dotatate treatment. Of these interviews, one person was interviewed twice on his initiative during and after the treatment cycle. The patients were between 58 and 82 years old, three of them were women, two men.^2^ The interviewees were diagnosed with NET between 2008 and 2020. They thus have been living from 1, 3, 4, 7 to 13 years with this diagnosis, in average 5.6 years (Median: 4). A seventh interview took place with a member of a NET patient organization. Of these seven interviews, two were conducted during the treatment in the patient’s room before injection, two in the hospital, and three via telephone. All interviews were carried out in French, recorded, transcribed verbatim, and translated into English.

The interviews were inductively coded following a Grounded Theory approach [35]. Thus, the codes were developed from the empirical material and, step by step, grouped into categories of higher abstraction. Three of the authors engaged with the empirical data and reflected their inductive findings through several online meetings. We continued this process throughout the whole data and reached theoretical saturation for several phenomena that were repeatedly confirmed throughout the analysis. Coding was performed using MAXQDA® software. The structure of the results section is shaped by both the interview guideline and the categories developed throughout the selective and axial coding phases.

Furthermore, one of the authors did several on-site visits in a specialized center in a French city, where she toured the facilities and observed the morning treatment of NET patients. Knowledge developed through these on-site visits were documented in an observation report, informed our coding, and supported the theoretical saturation by confirming key phenomena outlined in the results section.

Through the analysis of the data collected within the research project three dominant themes emerged: (1) the path to targeted treatment, (2) challenges of radiation and isolation, and (3) patient perceptions of healthcare delivery and care.

## Results

### The path to targeted treatment

All NET patients that we interviewed reported a long history of the disease before they received radionuclide therapy. All of them describe years of living with NETs, including different diagnoses and treatments, with varying degrees of success. Some patients got no or wrong diagnoses before a NET was detected, such as food intolerance, which causes similar side effects. Interviewee 1 described how gastroscopy and ultrasound did not result in any diagnoses and that she thought she had gluten intolerance. When she again developed shortness of breath, X-rays of the lung did not show any anomalies. When the patient started losing weight, her gastroenterologist advised her to eat gluten again to gain weight. Another blood test did not show any noticeable problems. It took some more time until she was able to get the right diagnosis.

Once a NET was detected, most patients faced years of established treatment, including surgeries and systemic therapy. This eventually turned out ineffective for most of them, as Interviewee 2 describes: “we did the check-ups and realized (…) it wasn’t working terribly, it wasn’t even working at all.” Another patient (Interview 3) received radionuclide therapy 11 years after her first chemotherapy. She described this treatment as the first one that showed positive results. Interviewee 4 had surgery in the mid-2010s, but the tumor could not be removed from the pancreas as it was close to an artery. The patient had chemotherapy for another 5 years but said that they “didn’t work too well” and that “the side effects were complicated.” He described that his oncologist expressed concerns about the side effects: “we can’t go on with chemo anymore (…), we have to find something else.”

To find out about and to consider radionuclide therapy, which might be offered only in a clinic far away from home, personal contacts, knowledge, and active engagement of patients in finding information about possible treatments are key. While one patient (Interview 2) described that she did her own research to find treatment, other patients had family, friends, and doctors, who played a crucial role in finally finding their way towards SRT treatment. Interviewee 5 learned about SRT through an acquaintance. She was offered chemotherapy in a hospital in another city in France and when she asked about radionuclide therapy, doctors continued to suggest chemotherapy as they did not know this other treatment option, as she reports:

I asked if I could do this nuclear therapy, and of course they didn’t agree because they didn’t know what it was. So, they had doctors’ meetings, they didn’t want to, they just wanted me to do chemo. (Interview 5)

This patient was even willing to travel to a neighboring country at her own expense because the specific treatment she was looking for did not seem available in France. However, her husband happened to know a pharmacist in another French town who told him that targeted therapy was practiced at a local clinic. She described that her surgeon—unlike some other oncologists—was open to new therapies and that he contacted a doctor and requested the treatment, which was finally accepted. The patient gained access to treatment, although she had to travel a long distance by train or car for it. She managed this with the help of various social contacts.

### Challenges of radiation and isolation

SRT involves radioactive material, which poses hazards for patients, healthcare professionals, and other contact persons. Its use requires different forms of social isolation that pose additional challenges. Throughout the interviews, patients described often having negative associations with radioactive material and expressed that its use for treatment feels unusual to them: “They explain to you that they’re going to put nuclear energy in your body (…) and that they’ve always told you that nuclear energy causes cancer and that they’re going to cure you” (Interview 6). This interviewee also reported that people with whom he talked about the use of radioactive material in SRT were at first confused because of their negative associations with radioactivity. In a previous interview, he explained how he associated radioactivity negatively with atomic bombs and nuclear power plants. In this earlier interview, he emphasized that his trust in science led him to accept the treatment:

I’m not a nuclear fanatic [laughs]. But finally, from what was explained to me, there is no other solution. When people are bombed with nuclear power, it doesn’t do them any good. Afterwards, it also generates mutations, things like that. But now what I suppose is that it has been carefully studied, carefully dosed, and that the effects, that the risk balance is positive, because otherwise I would not be offered the treatment. So, in the end, whether it’s chemo or nuclear, I don’t know if I prefer chemo or nuclear. (Interview 2)

While chronic (rare) diseases, in general, impact people’s social lives and may lead to a reduction of social contacts (see the “Research on patients with neuroendocrine tumors” section), this is particularly the case with SRT, since the precautionary actions required from patients increase social isolation. One patient described how social contacts were reduced as they are “not being allowed to receive visitors because it is a department that is completely closed to radiation” (Interview 5). Interviewee 4 emphasized: “You are isolated, you’re completely isolated, you don’t go out at all. You’re isolated, no–no, you don’t see anyone.” This patient thoroughly explained his activities during and some days after the treatment to avoid contamination of his surroundings with radioactivity. Healthcare professionals at the hospital asked him to separate his belongings, clothes, and bedsheets in boxes, as these may be contaminated with radioactivity, as she mentioned: “The room is sealed so we are not allowed to go out. You must put everything in different bags, food, clothes” (Interview 4).

Also, patients reported being advised to avoid social contact after the treatment due to radiation exposure. Interviewee 4 described how he was told to avoid leaving home, visiting stores, and to be particularly careful with children and pregnant women by staying away from them, as radioactivity decreases with distance. Another patient described how he managed these precautionary actions by avoiding contact:

Not meeting somebody the next week and the next three days, being careful, not seeing grandchildren, not meeting pregnant women. […] I saw no one, I was alone. I was careful not to be in contact, it was at least a week, with close people. I live by myself, I’m by myself so it wasn’t difficult at all (Interview 6)

Social isolation is not only given due to protective measures linked to radioactivity but also due to the time resources needed to undergo the treatment. SRT is also time-intensive and often requires traveling, as clinics are not always close to patients’ homes. This can be a burden for them, as one person explained:

I’m tired right now, with the trips and everything. Because I can no longer travel by plane because there is no longer a direct flight. […] I make the trips by train. […] The last time, I was almost eleven hours in the train to come back, so it’s really tiring. My back hurts. (Interview 5)

Since the treatment requires patients’ regular and prolonged absence from home, they cannot take care of other duties, such as caring for relatives. One patient described how she worries about her elderly husband when she is away, but that a neighbor and a cleaning person looked after him (Interview 4). Additionally, some interviewees reported administrative steps related to travel and social insurance.

### Patient perceptions of healthcare delivery and care

All respondents expressed satisfaction with SRT and how it was administered, and spoke predominantly positively about the involved hospital personnel, such as physicians, physicists, and (assistant) nurses. Many patients seemed to feel well overall during interviews and the site visits despite their vulnerable situation due to their disease and the treatment.

A key aspect that several patients mentioned during interviews and that may contribute to this positive sentiment relates to communication and social contacts with hospital staff. According to interviewees, physicians took their time to explain the treatment and the involved procedures. Various interviewees emphasized that the physicians were present, also during injections and that they offered availability for questions, as Interviewee 1 emphasized: “When I need something, a question to ask him, […] I do it by email and he answers me very quickly, and that’s great to be very reactive with his patients.” Interviewee 6 similarly explained how questions were answered in a short space of time: “within 24 or at worst 48 h.” They also described the importance of being able to expect an answer, as one patient states:

If there were any problems, I could always call Dr. B., who is very available for that, and who answered every phone call and e-mail. Of course, you shouldn’t abuse it, but he answers. As soon as you ask for something, it is sure that it will not remain unanswered. (Interview 5)

The responsiveness of doctors is furthermore linked to questions of confidence by another patient:

She is a person who knows how to put people at ease, who explains things from A to Z, and if you have a question to ask, she has the answer. She is very open to all questions, (…) she gives confidence. (Interview 3)

Indeed, several patients mentioned trust and confidence quite explicitly and linked these to responsiveness and their personal relationships with their doctors. One patient spoke about how trust between her and the doctors has been there for a long time, emphasizing that: “there is a history of trust between the doctors and me” (Interview 5). Another interviewee said: “I trust Dr. A. is someone who explains a lot, who says things as they will happen” (Interview 1). Another interviewee put this quite similarly and linked confidence with openness for explaining the treatment: “I had absolute confidence in Mrs. H. who had explained everything very, very well” (Interview 3). The same patient connects this to a new generation of medical doctors, which according to her are much more open to patients than doctors used to be. In line with that, she recalls experiences from earlier times when doctors were not as responsive: “they didn’t listen to anybody” (Interview 3).

Care of medical staff goes beyond the availability and provision of information of those conducting the SRT. Interviewee 5 met several assistants, nurses, and doctors, and she knew all of them by name. Patients repeatedly called staff members by their first or second names which emphasizes their emotional connection towards them. Several patients described the importance of social exchange, referring to confidence and positive emotions, as one patient said: “With Mrs. C., I saw the sun light up, so I said: it’s good, I’m not afraid anymore. She immediately put me at ease, and I felt good. I said: this will work” (Interview 4). Again, this might be fuelled by the rarity of the disease, the social isolation SRT treatment requires, and the specific care requirements that may facilitate more involvement, and personalized, close care from medical personnel. One patient emphasized her positive relationship with one doctor, which also includes the way how she informed her about medical updates and her treatment:

I was very happy because with Dr. A. it changed my medical world. (…) with Dr. A., it’s heaven! First, she explains everything we do, why we do it and so on. Then we have a human sense of relationship. And that’s it. It’s life-changing, you know. (Interview 2)

Furthermore, the notion of care was described in very literal ways. Patients report that the overall hospital management and general staff create an environment that makes the time at the clinic pleasant, given the circumstances. Sometimes this referred also to aspects outside the clinic, such as the organization of the commuting to the hospital, as one patient said: “it’s taken care of, I’m lucky” (Interview 2). Yet, most of the time, patients referred to practical and rather mundane aspects of care in the hospital, such as being offered an extra blanket for the night:

We are well taken care of; we are listened to. If I ask for an extra blanket, they give it to me. […] When you weigh 46 kg you are colder than when you weigh 54 like now. […] they were always responsive. (Interview 1)

Another patient (Interview 3) described that she was regularly unable to eat the hospital meals and that staff members managed, even at unusual eating times, to get different food she tolerated better. This patient also emphasized that the staff members did a great job despite a lack of available personnel. Interviewee 4 had to travel a long time to get treatment and was offered the opportunity for several examinations in 1 day, so he did not need to come in on several days. Interviewee 5 described how she was asked for her favorite music, which was then played during lengthy and regular MRI scans. The same patient emphasizes how positive the service and the staff were, how she and her illness were taken seriously, and that even though she was isolated in the unit, she did not feel alone, but rather welcomed and comfortable:

A service that is extraordinary, I want to emphasize that. The care workers and nurses are adorable, I must say that if it wasn’t for therapeutic purposes, I would have gone there on vacation! […] The people are really concerned about your health […]. While not being allowed to receive visitors, because it is a department that is completely closed to radiation, but you feel very well received. I know that every time it is like a big family. At least for me. I felt like that. With very nice people. (Interview 5)

## Discussion

The low prevalence of rare diseases is known to result in scarce resources, ranging from a lack of available expertise to appropriate care to patients [26]. NETs are typically classified as a rare disease and as such they share these challenges. Our research aligns with other studies [24, 28, 33] highlighting that some needs of NET patients are not met, especially when it comes to receiving a timely and appropriate diagnosis (see the “The path to targeted treatment” section). Furthermore, we find that notions of care, especially in the clinical setting of the SRT, are relevant to increasing patient well-being and their trust in medicine.

This paper shows that the NET patients interviewed are active seekers of care and that social and economic aspects matter. We already emphasized that this study conceptualizes care beyond nursing, encompassing all practices related to the needs and experiences of patients and caregivers [13]. For example, the patient trajectory of Interviewee 5 showed how social contacts make a difference and how she had to insist to get SRT, as it was unknown even to professionals. This circumstance links to the rareness of NETs and the selective application of SRT, given the complexity of its institutional embedding. However, actively pursuing care is easier for people with more social and economic resources. Insurances do not always cover travel costs, and two interviewed patients even considered treatment in other countries. Some interviewed patients engaged in a lot of effort to get the treatment they needed. But this is not possible for everyone, and thus easier for people with more social, cultural, and economic capital [36].

This inequality calls for more information and access to different treatment options. Indeed, like our interviewees, other studies show that a significant number of NET patients reported negative experiences with the explanations they received about their disease [37]. Kolarova and Bouvier [38] show that data on patient support groups for NETs is sparse but that there are some groups evolving which foster patient awareness and education, as well as access to relevant regulatory discourses regarding access to treatment and questions of care. The organizations mentioned in the “Research on patients with neuroendocrine tumors” section—the Neuroendocrine Tumour Research Foundation [21] and the Healing NET Foundation [22]—are just two examples of this development. By pointing towards the importance of groups like these, Kolarova and Bouvier [38] mirror strands of academic debates, which have studied the interactions between patients, patient organizations, and the medical realm [5, 7, 8], and have shown how patients actively engage with the medical system in a variety of ways.

SRT using 177Lu-Dotatate is one promising pathway for better health outcomes for NET-patients. While its application is still not fully standardized and controversial among experts, there are also hopes among some scientists for this to be a “magic bullet” against this type of cancer [3]. However, the metaphor exaggerates the known benefits of SRT and neglects known challenges and risks that professionals and patients need to deal with. This involves questions on balancing health outcomes and risks of radiation [15, 39], questions of institutional embedding and management [40], and the need for interdisciplinary collaboration [41]. When addressing emerging technologies, there is a general tendency in science towards a technology-oriented risk-and-benefit perspective, while focusing less on relevant social and ethical aspects [42].

A technology-oriented perspective also dominated the POPEYE project, from which this article emerged. This is in line with a general epistemic shift towards molecularization and personalization in cancer treatment [43], which implies that there is an evolving therapeutic landscape that has shifted towards molecularly targeted therapies, allowing for tumor-agnostic personalized medicine. A major concern of POPEYE is the optimization and individualization of applying 177Lu-Dotatate through advanced imaging and dosimetry. The field of radiomics, including SRT, has been growing in the past decades, including dosimetry simulations that rely on machine learning tools [44]. There are hopes to manage radiation risks through such simulations, also by personalizing treatment and thus lowering radioactive doses delivered to the patients’ bodies in the future [3, 45]. However, such approaches are not free of errors. Simulations often rely on non-transparent (in-house) algorithms and their application is non-standardized across hospitals [40]. Yet, these questions, which are important for scientists and health care professionals, were not the concerns of any of our interviewees expressed. Thus, besides advancing treatment options for future patients through research and innovation, it is important to explore and account at the same time for the present needs of patients [4].

All interviewed patients emphasized the importance of feeling well cared for by their healthcare providers. While communication with and trust in medical personnel have been described as relevant aspects in studies on various diseases [7, 11], it may be particularly relevant in the case of SRT, due to social isolation needs because of radiation. Our interviewees were mainly concerned with the care they experienced at the hospitals, which was, in their stories, mainly tied to exchange with medical personnel. Some patients mentioned care, in the form of good coordination and communication, as an explicit factor for trust and confidence in a treatment that involves the injection of radioactive material to the body. The interviewees expressed how they value that several doctors and caregivers considered not only their illness but, moreover, their overall wellbeing and that they understand the procedures. This points to the well-known importance of empathic engagement of medical staff with patients as well as possibilities and time resources for meaningful interaction [7]. These may be particularly important in the case of SRT, given the radiation risks, time intensiveness, and the challenge of social isolation.

Yet, there are some limitations that must be considered in these results. We present data of a relatively small sample of interviewees from the French context. While the interviews provide rich and in-depth insights, the overall amount of seven interviews must be considered for these findings. Furthermore, there might be a bias in our sample as those in good health may be more willing and able to give interviews. Given the sampling through hospital personnel, we might have also recruited persons who are more satisfied with the treatment overall. The number of interviewees must yet be reflected in light of the overall low prevalence of patients, even at the national level. Some of the hospitals which the patients were referred to only treat around ten cases a year, which explains why this research builds on comparably few in-depth cases. Additionally, field access was challenging not only due to the health conditions of the patients and the precautionary measures at the nuclear departments, but also because our research was conducted during the COVID-19 pandemic.

Our study highlights the comprehensive needs of patients in a specific case of an emerging technology in cancer treatment. It also demonstrates the necessity to improve access to SRT, especially for patients who are less able to actively seek out specific treatment. The challenges associated with SRT are related to various patient care needs that go beyond nursing and receiving medical treatment. Thus, further transdisciplinary research that explores patients’ perspectives could help to increase their well-being and trust in the medical staff.

## Data Availability

No datasets were generated or analysed during the current study.

## Footnotes

1 The POPEYE (Personalized Optimization of Prognostic and thErapeutic protocols with Lu-177 for MNETs, through the development of advanced computational tools and a portable detection sYstEm) project was conducted from 2020 to 2023.

2 Given the overall low number of patients receiving SRT in France, socio-demographic data are not displayed in detail to protect individual privacy.

## Declarations

### Other identifying information

There is no other identifying information.

They confirm that the manuscript is comprised of original material that is not under review elsewhere, and that the study on which the research is based has been subject to appropriate ethical review.
